# A 29-Year-Old Female with Progressive Myoclonus and Cognitive Decline

**DOI:** 10.1155/2013/125672

**Published:** 2013-04-11

**Authors:** D. Taylor, H. R. Haynes, A. Graham, S. Gerhand, K. M. Kurian

**Affiliations:** ^1^Department of Medicine, Bath Royal United Hospital, Bath BA1 3NG, UK; ^2^Department of Neuropathology, Frenchay Hospital, Bristol BS16 1LE, UK; ^3^Department of Neurological Rehabilitation, Frenchay Hospital, Bristol BS16 1LE, UK; ^4^Department of Neuropsychology, Frenchay Hospital, Bristol BS16 1LE, UK

## Abstract

Myoclonic epilepsy with red ragged fibres (MERRF) is a rare mitochondrial disorder presenting with progressive myoclonus, epilepsy, and cognitive decline. Here, the authors present a case of a 29-year-old lady presenting with myoclonus and describe the subsequent investigations that led to a diagnosis of MERRF. In addition, we examine her cognitive decline over a 9-year period, demonstrating a feature commonly seen in mitochondrial cytopathies.

## 1. Background

Myoclonic epilepsy with red ragged fibers (MERRF) is a rare mitochondrial disorder associated not only with progressive myoclonus and epilepsy but also with cognitive and functional decline in a young age group [1, 2]. The neurodegenerative nature of MERRF is demonstrated here with comparative neuropsychological testing of a 29-year-old female over a 9-year period which shows changes consistent with dementia.

## 2. Case Presentation

A previously well 29-year-old lady presented to neurology services in 2003 complaining of daily “jerking” of one or more limbs. This was associated with intermittent tremulous episodes and 18-month history of twice-weekly migraine with visual aura. She described a right-sided headache, vice-like in nature, which was progressive and worse on waking. Exercise appeared to precipitate presyncopal symptoms and a feeling of detachment from her surroundings. Admission was triggered by a generalized tonic-clonic seizure. There was no reported change in memory, cognition, or coordination. She took the oral contraceptive pill and family history included an uncle with multiple sclerosis. She was an only child and had no children of her own. She was completely independent with activities of daily living.

Examination demonstrated visual acuity of 6/9 in the right eye and 6/18 in the left eye. Pupils reacted equally to light and accommodation and there was no relative afferent pupillary defect or deficiency on Ishihara testing. Ophthalmoscopy revealed healthy retinae bilaterally with slightly hypopigmented choroid felt to be in keeping with the patient's complexion. No opthalmoplegia was present and there was no deficiency in facial sensation or movement. No conductive or sensorineural hearing loss was found and she demonstrated normal tongue and palatal movements. 

There was no evidence of tremor or myoclonus on examination of the upper and lower limbs, with no evidence of muscle wasting. She had normal muscle tone and power in all muscle groups. Reflexes were normal in the upper limbs, yet she appeared globally hyperreflexive throughout the lower limbs with a crossed adductor reflex. Plantar responses were flexor. Sensory testing was normal in all modalities with no gait or truncal ataxia. There was minimal left upper limb ataxia with some slowness when doing up buttons. Speech was normal and there were no other signs of cerebellar dysfunction.

Examination of other systems was unremarkable. 

Initial neuropsychological assessment in 2003 revealed no significant cognitive dysfunction scoring in the average range on testing verbal comprehension, perceptual organisation, working memory, and processing speed.

## 3. Investigations 

**Figure 1:**
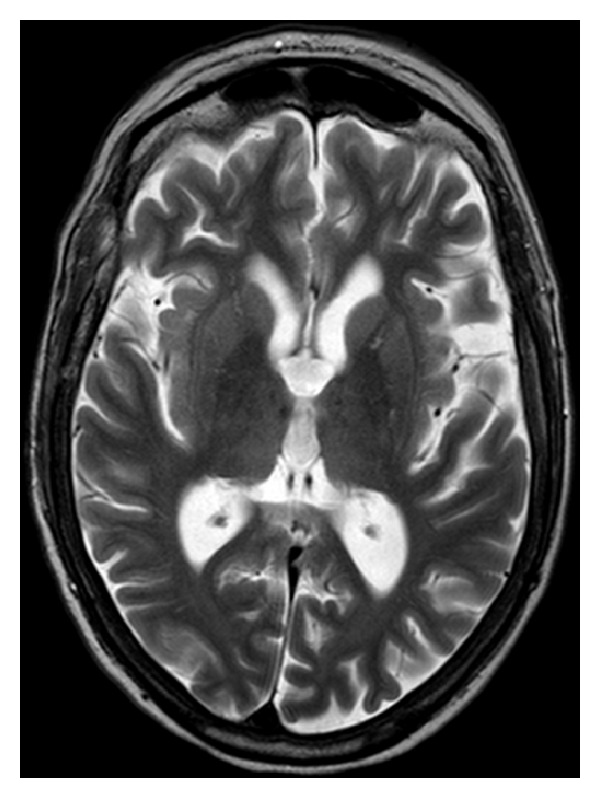
MRI brain showing CSF spaces and cerebral atrophy that are prominent for a patient of this age.

Blood testing including a vasculitic and autoimmune profile was normal. Lumbar puncture showed a normal opening pressure and routine CSF examination was clear. CSF S100b protein was slightly elevated (suggesting generalised CNS astrocytosis) [3]. CSF 14-3-3 protein was normal [4]. An EEG showed nonspecific generalised brain dysfunction and an MRI brain revealed diffuse cortical atrophy with prominent CSF spaces for the patient's age (Figure 1).

**Figure 2:**
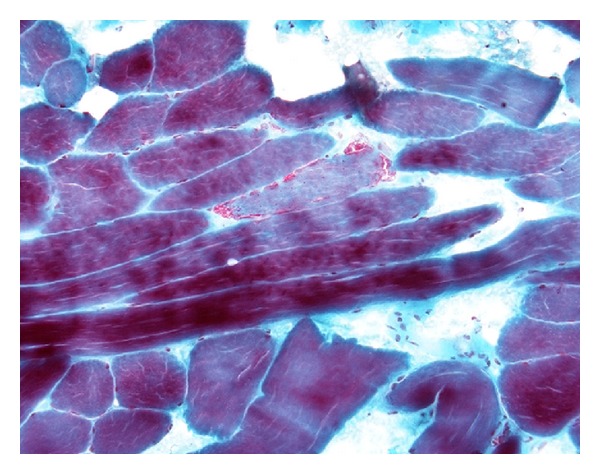
Muscle biopsy (modified Gomori trichrome) showing moderate numbers of fibrils with a red-ragged appearance with accumulation of red reaction product in the subsarcolemmal region and between myofibrils.

**Figure 3:**
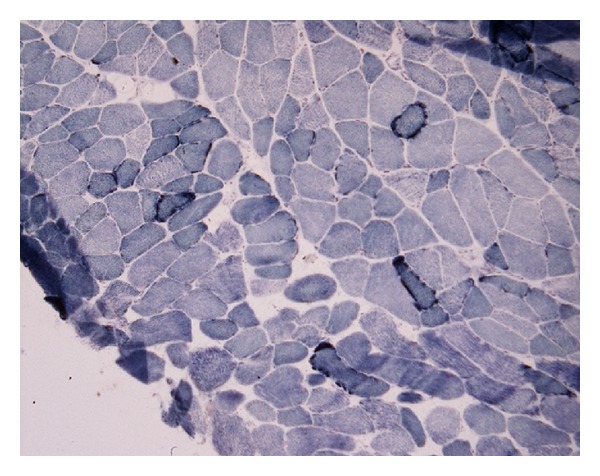
Increased staining “ragged blue fibres” on succinate dehydrogenase preparation.

A muscle biopsy was performed which had appearances consistent with a mitochondrial cytopathy with ragged red fibres (Figures 2 and 3).

Serum genetic testing was undertaken: this tested negative for Friedreich's ataxia, dentatorubral-pallidoluysian atrophy (DRPLA), and the spinocerebellar ataxias but was positive for the mitochondrial A83445 mutation. This finding in combination with the muscle biopsy and patient's symptoms diagnose myoclonic epilepsy with red ragged fibres (MERRF) [5].

## 4. Outcome and Followup 

The patient was readmitted to hospital in 2012 following a further tonic clonic seizure (her first since diagnosis in 2003). The frequency of myoclonus had been steadily increasing and she had become increasingly dependent. Repeat neuropsychological testing showed a global deterioration in cognitive function compared with previous assessment. There was a marked decline in perceptual reasoning and impairment in immediate and visual working memory, along with deterioration in language and executive function. This was consistent with dementia.

## 5. Discussion 

The mitochondrial disease MERRF is a rare (prevalence at least 1 : 10,000 [6]) multisystem disorder with clinical features including progressive myoclonus, myopathy, generalized tonic-clonic seizures, ataxia, neurosensory deafness, and progressive cognitive impairment [2]. 

Diagnosis of MERRF relies on typical symptoms of myoclonus, epilepsy, and ataxia along with findings at muscle biopsy. The increased red staining at the subsarcolemmal and intermyofibrillar region with Modified Gomori Trichrome preparation represents accumulations of morphologically abnormal mitochondria, some of which will have paracrystalline inclusions on electron microscopy. Succinate dehydrogenase activity may also be increased in the ragged red fibres.

In addition, neuropathological examination of the cerebral cortex reveals neuronal loss, astrocytosis, and degeneration of myelinated tracts [7]. The cerebellum is preferentially affected, with neuronal degeneration in the dentate nucleus. Significant reductions in neurons in the inferior olivary nucleus as well as the gracile and cuneate nuclei and Clarke's column in the spinal cord may also be seen [8].

The diagnosis is supported by genetic analysis for the common mitochondrial DNA translocation mutations: A8344G (found in 80% of patients), A3243G, and T8993C/G [5]. These mutations alter the intramitochondrial synthesis of the 13 mtDNA (mitochondrial DNA) encoded mitochondrial respiratory chain proteins which in turn lower intracellular ATP. This decreases neuronal membrane potential and leads to excitotoxic lesions and epilepsy [9]. 

MERRF is one of the spectra of mitochondrial disorders with frequent seizures that include Alpers-Huttenlocher, ANS spectrum (MIRAS and SANDO), Leigh syndrome, MELAS, and MSCAE [10]. The differential diagnosis should also include metabolic/storage disorders, prion disease, and any slow virus of the central nervous system. 

Treatment of mitochondrial disorders is supportive with anticonvulsants used to control seizures and myoclonus; however, patients with MERRF may have refractory symptoms which can develop into continuous generalised myoclonus. Trials of coenzyme Q10 to improve muscle strength are currently without a strong evidence base [11]. 

Patients with MERRF tend to be young and of child bearing age and so genetic counselling should be offered. MERRF is maternally inherited; however, the proband's mother may or may not clinically express the disease. All offspring will inherit the mtDNA from an affected mother. To what extent they will clinically express disease and at what severity are impossible to predict at this time [1].

This case highlights well the progressive nature of myoclonus and cognitive decline seen in MERRF over a 9-year period. Neuropsychological assessment demonstrated a clear cognitive deterioration with changes consistent with dementia.

## 6. Learning Points

 Mitochondrial cytopathies such as MERRF are associated with progressive cognitive decline.
 Diagnosis of MERRF relies on typical symptoms of myoclonus, epilepsy, and ataxia along with typical histological findings at muscle biopsy.
 The diagnosis is supported by genetic analysis of mitochondrial DNA where 80% of patients will carry an A8344G mutation.
 Treatment of mitochondrial disorders is supportive.

## Abbreviations

ANS: Ataxia neuropathy spectrum
MIRAS: Mitochondrial recessive ataxia syndrome
SANDO: Sensory ataxia, neuropathy, dysarthria, and opthalmoplegia
MELAS: Mitochondrial encephalopathy, lactic acidosis, and stroke like episodes
MSCAE: Mitochondrial spinocerebellar ataxia and epilepsy.
